# Normobaric Oxygen May Ameliorate Cerebral Venous Outflow Disturbance-Related Neurological Symptoms

**DOI:** 10.3389/fneur.2020.599985

**Published:** 2020-11-13

**Authors:** Jiayue Ding, Yu Liu, Xiangyu Li, Zhiying Chen, Jingwei Guan, Kexin Jin, Zhongao Wang, Yuchuan Ding, Xunming Ji, Ran Meng

**Affiliations:** ^1^Department of Neurology, Xuanwu Hospital, Capital Medical University, Beijing, China; ^2^Department of Neurology, Tianjin Medical University General Hospital, Tianjin, China; ^3^Epilepsy Center, Beijing Fengtai You'anmen Hospital, Beijing, China; ^4^Department of Neurology, Tianjin Huanhu Hospital, Tianjin, China; ^5^Advanced Center of Stroke, Beijing Institute for Brain Disorders, Beijing, China; ^6^Department of China-America Institute of Neuroscience, Xuanwu Hospital, Capital Medical University, Beijing, China; ^7^Department of Neurosurgery, Wayne State University School of Medicine, Detroit, MI, United States; ^8^Department of Neurosurgery, Xuanwu Hospital, Capital Medical University, Beijing, China

**Keywords:** normobaric oxygen, cerebral venous outflow disturbance, brain dysfunction, EEG, neurological impairment

## Abstract

Cerebral venous outflow disturbance (CVOD) has begun to garner the attention of researches owing to a series of clinical symptoms that impose a significant impact on people's quality of life. Herein, we aimed to investigate whether normobaric oxygen (NBO) can ameliorate CVOD-induced neurological symptoms. This was one part of the prospective trial registered in ClinicalTrials.gov (NCT03373292). A total of 37 CVOD patients were divided into the NBO group (5–8 L/min of oxygen inhalation, 1 h per time, 3 times daily, *n* = 19) and the control group (without oxygen inhalation, *n* = 18) randomly. The assessments were performed at admission, 1-week hospitalization, and 6-month follow-up. Quantitative electroencephalogram (qEEG) data were recorded prior to and post 1 h of NBO in some patients. R software was used for data analysis. No NBO-related adverse events were observed during the whole NBO intervention process. The 1-week Patient Global Impression of Change (PGIC) scale showed that the symptom improvement occurred in nine patients in the NBO group (47.4%) while none in the control group (*p* = 0.001). NBO could improve headache evaluated with visual analog scale (pre-NBO vs. post-NBO: 4.70 ± 2.16 vs. 2.90 ± 2.03, *p* = 0.024) and Headache Impact Test-6 (53.40 ± 12.15 vs. 50.30 ± 13.04, *p* = 0.041). As for 6-month PGIC follow-up, eight out of 14 cases (57.1%) in the NBO group reported improvement, while only one out of 12 patients in the control group replied mild improvement (*p* = 0.014). The qEEG revealed that NBO reduced the ratio of theta to alpha power (0.65 ± 0.38 vs. 0.56 ± 0.35, *p* = 0.030) over the fronto-central electrodes. To sum up, NBO may be a safe and effective approach to attenuate CVOD-related symptoms (especially for headache) by brain functional improvement resulting from increasing oxygen supply to the brain tissues.

## Introduction

Recently, a growing body of literature has demonstrated the causative role of impaired venous drainage function or structures on the intractable neurological deficits, in which cerebral venous sinus stenosis (CVSS) and internal jugular venous stenosis (IJVS) are in the majority (1–7). No matter what kinds of the cerebral venous outflow disturbance (CVOD), long-term elevated trans-stenosis pressure gradient leads to venous outflow disturbance and further affects the whole arteriovenous circulation, resulting in cerebral circulation insufficiency (CCI) consequently (1–7). A variety of associated clinical symptoms, such as headache, tinnitus, head noise, insomnia, dizziness, and even high intracranial hypertension (IH), impose a significant impact on people's well-being and quality of life (3, 4, 8, 9). Endovascular stenting may be a promising regimen against CVOD; however, given the technical constraints, it is not very commonly used in clinical settings (10–12). Importantly, most of the patients with osseous impingement-induced venous stenosis are inappropriate for stenting (2–4). Moreover, a proper specific stent for cerebral venous sinus (CVS) and internal jugular veins (IJV) is still lacking, and notably, this method is under exploration period currently. A convenient, safe, and effective adjuvant therapy is warranted to improve the venous anomaly-related symptoms.

Normobaric oxygen (NBO), supplied by a face mask (such as simple mask) with one atmosphere pressure (1 ATA = 101.325 kPa), is a routine adjuvant therapy for various diseases, especially for brain ischemia (13–15). NBO can elevate brain tissue oxygen pressure (pO_2_), increase cerebral blood flow and volume (CBF and CBV), protect the blood–brain barrier (BBB), attenuate ischemic injury, and ameliorate inflammation in CCI (16–21). Similar to cerebral arterial disease-induced CCI, CVOD also induces CCI by reducing CBF, damaging the BBB, promoting inflammatory factor release, and facilitating neuron injury (1–7), whereby NBO may be capable of yielding some benefits to patients with CVOD as well (2, 3, 7). This study aimed to investigate the safety and efficacy of NBO on correcting CVOD-related neurological symptoms to explore an adjuvant therapy for CVOD.

## Materials and Methods

### Subjects

This study was an assessor-bind randomized controlled study, which was one part of the prospective trial registered with ClinicalTrials.gov (NCT03373292). It had been approved by the Institutional Ethic Committee of Xuanwu Hospital, Capital Medical University (Beijing, China) based on the guidelines of the 1964 Declaration of Helsinki. Informed consent was obtained from all individuals before any specific interventions or tests performed. It has been acknowledged that 10–20 patients per group are often adequate for determining whether a larger multicenter trial should be conducted (22, 23). Therefore, we aimed to enroll 15–20 patients in the protocol, and the sample size might be changed in accordance with the funding.

Cerebral venous outflow disturbance consisted of CVSS and IJVS, which referred to the narrowing degree of the inner diameter of IJV or CVS reached ≥50% in respect to the proximal adjacent IJV or CVS segment; meanwhile, with one abnormal collateral vessel, the inner diameter of collateral vessel at least must be ≥50% of the maximal inner diameter of the adjacent IJV, or with two or more abnormal collateral vessels, the inner diameters of these collateral vessels were without special definition, as presented in magnetic resonance venography (MRV) and/or computed tomographic venography (CTV) (2–5, 24, 25). Since representing the severity of the venous outflow insufficiency, abnormal collateral generation is essential for diagnosis of IJVS and CVSS.

All of the patients complied with the inclusion and exclusion criteria and were enrolled after giving their informed consent.

Inclusion criteria were as follows: (1) age range from 18 to 80 years; (2) definite radiographic diagnosis of IJVS or CVSS; (3) suffered focal or non-focal neurological symptoms; (4) inappropriate for cerebral endovascular treatment; and (5) National Institutes of Health Stroke Scale (NIHSS) ≤ 4, modified Rankin scale (mRS) ≤ 2.

Exclusion criteria were as follows: (1) comorbid with other life-threatening diseases; (2) clinical symptoms can be illustrated by other diseases; (3) with a history of cerebral endovascular surgery; (4) confirmed as moderate or severe intracranial/extracranial arterial stenosis or with ischemic/hemorrhagic stroke history; and (5) poor compliance.

In order to identify the electroencephalogram (EEG) features for CVOD, a cohort of healthy volunteers were also incorporated into this study. This population should have confirmed without any cerebral vascular diseases and with no vascular disease-related risk factors (such as hypertension, diabetes, dyslipidemia, and autoimmunity disease).

### Interventions

**Figure 1:**
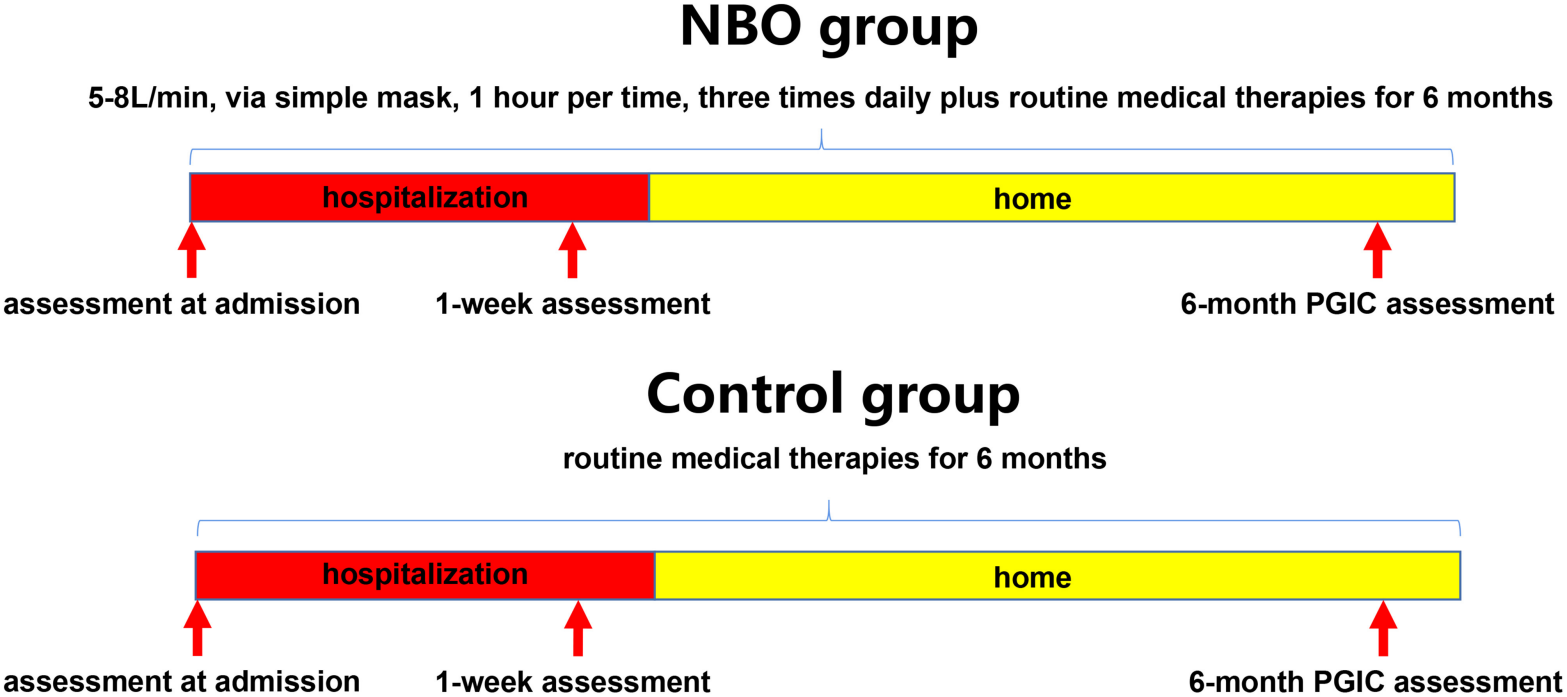
Study flowchart.

Patients were randomly assigned in a 1:1 ratio to receive routine medical therapies plus oxygen supplement or routine medical therapies alone by means of a random number table; the patients in the NBO group underwent 1 h of 5–8 L/min of oxygen inhaling via a simple mask, by oxygen concentrator each time, three times daily besides routine medical therapies for 6 months (1–2 weeks in a hospital); the patients in the control group only underwent routine medical therapies for 6 months (1–2 weeks in a hospital). Routine medical therapies included neuron nutrition, anti-inflammatory, anti-coagulation, and symptomatic treatments (Figure 1).

### Scale Assessment

Each of the enrolled subjects should fill in a questionnaire at admission to delineate the symptoms they had. They would blind to the proposed effectiveness of each treatment strategy in order to avoid subjective bias. According to the common non-specific neurological symptoms seen in the patients with CVOD, some scales were recorded at admission, that is, visual analog scale (VAS; 0–10 scores) and Headache Impact Test-6 (HIT-6; 36–78 scores) for headache; Athens insomnia scale (AIS; 0–24 scores) and insomnia severity index (ISI; 0–28 scores) for insomnia; tinnitus handicap inventory (THI; 0–100 scores) for tinnitus/head noise; and hospital anxiety and depression scale (HADS; 0–42 scores) for anxiety and depression (26–31). Higher scores mean severe symptoms. All of the above scales were recorded again at 1-week hospitalization. Additionally, the Patient Global Impression of Change (PGIC) scale was also assessed at this time (32). A phone call follow-up was performed in each patient at 6 months, and only PGIC scale was recorded due to the constraints of this follow-up method (Figure 1). The assessors were blind to the assignment when evaluating PGIC scale.

### Electroencephalogram Acquisition and Analysis

The EEG data in some patients were recorded with the EEG recording equipment (EEG YAL PN-NET, Beijing Yunshen Science and Technology Corporation). Ag/AgCl electrodes were positioned at Fp1, Fp2, Fpz, F3, F4, F7, F8, Fz, C3, C4, Cz, P3, P4, Pz, T3, T4, T5, T6, O1, O2, Oz, A1, and A2 (23 channels) according to the international 10–20 system, with electrode impedances all <5 kΩ. The sampling rate was 256 Hz for all channels using a 16-bit AD convertor. The healthy volunteers underwent 30-min EEG recording for one time, and the CVOD patients underwent 30-min EEG recording for two times. During the interval between the two times of recording, the patients underwent 1 h of 5–8 L/min of NBO via a simple mask. Firstly, compared with healthy EEG, the CVOD-related EEG anomalies would be determined. Secondly, the EEG datasets prior to and post-NBO treatment would be compared to investigate the impact of NBO on brain functions.

Five minutes of artifact-free data was selected from EEG recordings in each subject at random, and these participants should remain awake and lying quietly with eyes closed as much as possible. Channels A1 and A2 were the references. Visual artifact rejection and data filtering (band pass, 1–30 Hz) were performed in EEGLAB (version 2019.1) with supplementary scripts operating in the MATLAB environment (33). Meanwhile, artifact removal (blink artifacts and electromyogram artifacts) was also performed using BSS algorithm (sobi). The absolute power (AP; with units μV^2^) of the delta (1–4 Hz), theta (4–8 Hz), alpha (8–13 Hz), and beta (13–20 Hz) frequency bands was computed using fast Fourier transform for each electrode with a 2-s Hamming window length. The relative power (RP; the frequency band of interest power/the total power across the 1- to 20-Hz range), theta/alpha AP ratio (TAR), delta/alpha AP ratio (DAR), and (delta+theta)/(alpha+beta) AP ratio (DTABR) were calculated as well. Besides overall channels, the EEG oscillations over the fronto-central electrodes (C3, C4, Cz, F3, F4, and Fz channels) were also used to evaluate the patient's brain function due to the minimal occipital alpha oscillation contamination.

### Outcomes

The primary outcome was 1-week PGIC. Patient Global Impression of Change is a self-reported evaluation of symptom improvement by patients; it consists of a 5-point scale (1 = obvious improvement; 2 = mild improvement; 3 = no change; 4 = mild deterioration; and 5 = significant deterioration) (32).

The secondary outcomes include 1-week VAS, HIT-6, AIS, ISI, THI, and HADS scales, and 6-month PGIC evaluated through phone call follow-up. Meanwhile, the EEG features prior to and post-NBO intervention were also compared in order to assess the transient effect of NBO.

### Statistical Analysis

R software (http://www.r-project.org) was used for the analysis in this study. Continuous data following a Gaussian distribution were expressed as mean ± standard deviation (SD) and analyzed with Student's *t*-test; otherwise as median [interquartile range (IQR)] and analyzed with Mann–Whitney U-test. The comparison between prior to and post-intervention used paired samples *t*-test and Wilcoxon-test. Categorical data were presented as *n* (percentage) and preceded by Fisher's exact-tests. *p-*value < 0.05 was indicative of statistical significance.

## Results

### Patient Demographics

From January 2018 through June 2019, a total of 37 patients who were confirmed as CVOD in the Department of Neurology, Xuanwu Hospital, Capital Medical University, were recruited in this study, in whom 19 cases were labeled as the NBO group and 18 cases were called the control group. The baseline characteristics between the two groups showed no statistical difference. Details are displayed in Table 1.

**Table 1:** Baseline demographics of the enrolled subjects in the NBO group and the control group.

**Items**	**NBO**	**Control**	***p*-value**
Case number	19	18	NA
Female	9 (47.7)	12 (66.7)	0.325
Age, years	55.8 ± 15.4	53.2 ± 13.9	0.591
Smoke	1 (5.3)	4 (22.2)	0.180
Drink	1 (5.3)	1 (5.6)	>0.999
**Comorbid diseases**			
Hypertension	8 (42.1)	3 (16.7)	0.151
Diabetes	2 (10.5)	2 (11.1)	>0.999
Hyperlipidemia	6 (31.6)	6 (33.3)	>0.999
Hyperhomocysteinemia	2 (10.5)	2 (11.1)	>0.999
Hyperuricemia	1 (5.3)	1 (5.6)	>0.999
Hepatitis	3 (15.8)	3 (16.7)	>0.999
Comorbid disease free	7 (36.8)	8 (44.4)	0.743
**Imaging finding**			
Intracranial arterial stenosis	2 (10.5)	2 (11.1)	>0.999
**CVSS**			
Left CVSS	5 (26.3)	4 (22.2)	>0.999
Right CVSS	4 (21.1)	4 (22.2)	>0.999
**IJVS**			
Left J1	3 (15.8)	0 (0.0)	0.230
Left J2	2 (10.5)	2 (11.1)	>0.999
Left J3	12 (63.2)	6 (33.3)	0.103
Right J1	1 (5.3)	0 (0.0)	>0.999
Right J2	1 (5.3)	2 (11.1)	>0.999
Right J3	9 (47.4)	11 (61.1)	0.515
**Clinical manifestations**			
Time from symptom onset to enrollment, months	36.0 (12.0, 72.0)	54.0 (21.0, 126.0)	0.461
Insomnia	12 (63.2)	11 (61.1)	0.743
Hearing impairment	6 (31.6)	6 (33.3)	>0.999
Visual impairment	5 (26.3)	10 (55.6)	0.099
Headache	10 (52.6)	12 (66.7)	0.385
Tinnitus	9 (47.4)	6 (33.3)	0.508
Head noise	8 (42.1)	6 (33.3)	0.737
Dry eyes	11 (57.9)	11 (61.1)	>0.999
Uncomfortable neck	9 (47.4)	9 (50.0)	>0.999
Dizziness	11 (57.9)	11 (61.1)	>0.999
Anxiety or depression	10 (52.6)	8 (44.4)	0.746
Nausea or vomiting	3 (15.8)	4 (22.2)	0.693
Memory deterioration	4 (21.1)	4 (22.2)	>0.999
Medians (IQR) number of manifestations	5.0 (4.0, 8.0)	5.5 (4.0, 8.3)	0.916

*Continuous variates with Gaussian distributions are presented as mean ± standard deviation; otherwise, the variates are presented as median [interquartile range (IQR)]. Categorical data are expressed as n (%). NA, not available; NBO, normobaric oxygen; CVSS, cerebral venous sinus stenosis; IJVS, internal jugular venous stenosis; IQR, interquartile range*.

### Safety Outcomes

No NBO-related adverse events occurred during NBO intervention. All patients tolerated NBO treatment during hospitalization and follow-up time period. No major discomfort as a result of NBO intervention was reported.

### Primary Outcome

The 1-week PGIC showed that nine out of the 19 patients (47.4%) in the NBO group reported improvement (three cases with remarkable improvement, six cases with mild improvement, seven cases with no change, and three cases with mild deterioration), while no patients in the control group felt that their symptoms improved (11 cases with no change and seven cases with mild deterioration). The difference illustrated a statistical significance (*p* = 0.001).

### Secondary Outcome

There were 10 patients in the NBO group and 12 patients in the control group complaining of headache. According to the VAS, six patients in the NBO group (60.0%) reported headache ameliorated, while only one patient in the control group (5.6%) felt mildly improved headache (*p* = 0.020). Compared with the baseline, the VAS in the NBO group was significantly improved at 1-week investigation (4.70 ± 2.16 vs. 2.90 ± 2.03, *p* = 0.024); however, it did not change at 1 week in the control group (3.42 ± 2.39 vs. 3.50 ± 2.54, *p* = 0.754). Additionally, there was no difference of VAS between the NBO group and the control group at 1-week hospitalization (*p* = 0.553). Based on the HIT-6 scale, seven patients (70.0%) in the NBO group reported headache relief, while no one in the control group reported headache attenuation (*p* = 0.001). The 1-week HIT-6 scale was obviously better than their baseline in the NBO group (53.40 ± 12.15 vs. 50.30 ± 13.04, *p* = 0.041) but has no changes in the control (54.17 ± 11.98 vs. 55.00 ± 11.36, *p* = 0.096). However, the difference of the 1-week HIT-6 between the NBO group and the control group did not reach statistical significance (*p* = 0.377).

**Figure 2:**
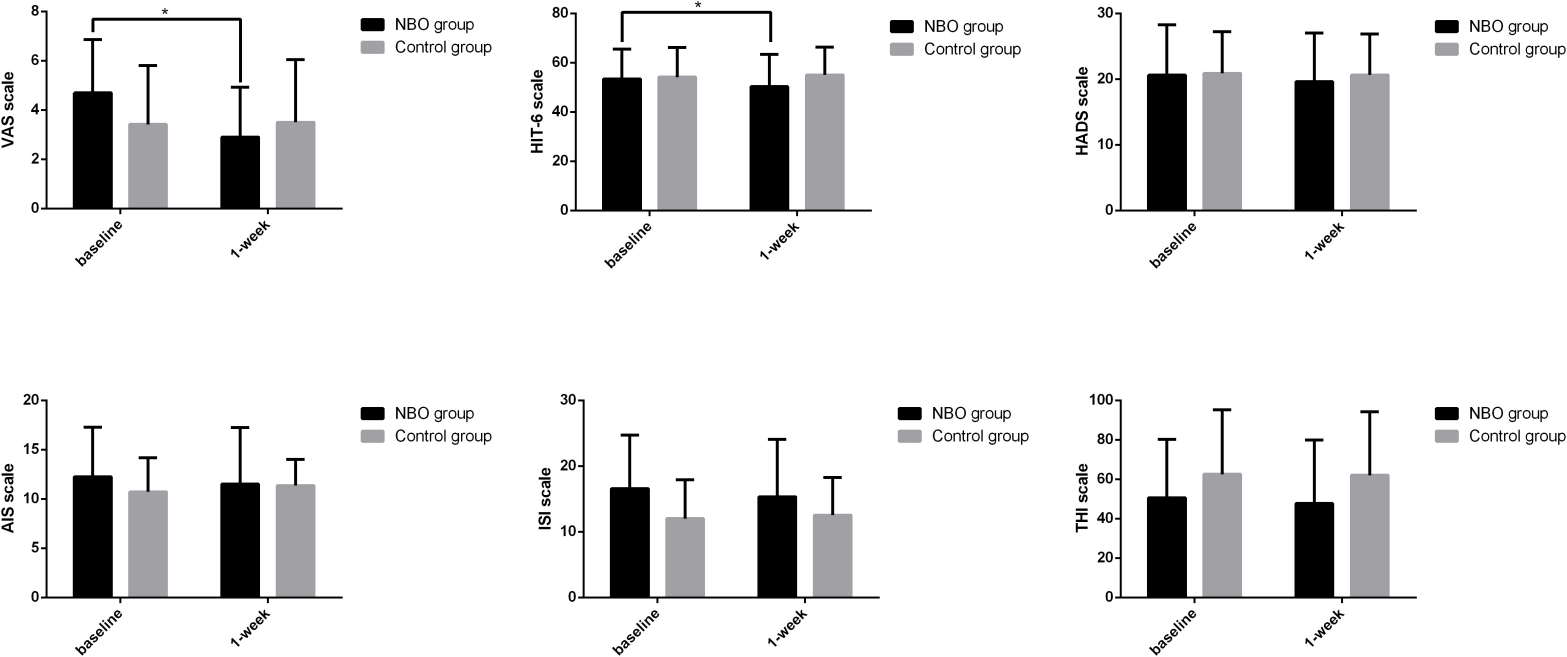
The symptom scales in normobaric oxygen (NBO) and control groups. Visual analog scale (VAS) and Headache Impact Test-6 (HIT-6) scale declined after 1-week NBO performance but remained unchanged after routine medical management. Compared with baseline, 1-week hospital anxiety and depression scale (HADS), Athens insomnia scale (AIS), insomnia severity index (ISI), and tinnitus handicap inventory (THI) improved in the NBO group; however, this change did not reach a statistical significance. *p < 0.05.

Other secondary outcomes including anxiety/depression assessed by HADS scale, insomnia assessed by AIS and ISI scale, and tinnitus/head noise assessed by THI scale showed no statistic difference between the NBO and control groups. The proportions of the improved patients in each group are presented in Table 2, and the scale scores are detailed in Figure 2 and Table 3.

**Table 2:** Clinical outcomes of CVOD patients in the NBO group and the control group.

	**NBO**	**Control**	***p*-value**
**1-week hospitalization**			
Case number	19	18	NA
Improved (PGIC)	9 (47.4)	0 (0.0)	0.001
Cases with anxiety/depression	10	8	NA
Anxiety/depression improved (HADS)	1 (10.0)	0 (0.0)	>0.999
Cases with headache	10	12	NA
Headache improved (VAS)	6 (60.0)	1 (5.6)	0.020
Headache improved (HIT-6)	7 (70.0)	0 (0.0)	0.001
Cases with insomnia	12	11	NA
Insomnia improved (AIS)	2 (16.7)	0 (0.0)	0.481
Insomnia improved (ISI)	2 (16.7)	0 (0.0)	0.481
Cases with tinnitus/head noise	15	7	NA
Tinnitus/head noise improved (THI)	1 (6.7)	0 (0.0)	>0.999
**1-year follow-up**			
Case number	14	12	-
Improved (PGIC)	8 (57.1)	1 (8.3)	0.014

*HADS includes three scales: 0–7, non-symptom; 8–10, suspected symptom; 11–21, definite symptom. VAS includes three scales: 0–3, mild headache; 4–6, moderate headache (causing insomnia); 7–10, severe headache. AIS includes three scales: 0–3, non-symptom; 4–6, suspected symptom; 7–24, definite symptom. ISI includes four scales: 0–7, non-symptom; 8–14, suspected symptom; 15–21, mild or moderate symptom; 22–28, severe symptom. The aforementioned scores decreasing one or more scales indicate that the symptom is improved. THI scores decreasing more than 20 scores indicate that the noise is ameliorated. HIT-6 scores decreasing more than 2 scores indicate that the headache is relieved*.

*CVOD, cerebral venous outflow disturbance; NBO, normobaric oxygen; PGIC, Patient Global Impression of Change; HADS, hospital anxiety and depression scale; VAS, visual analog scale; HIT-6, Headache Impact Test-6; AIS, Athens insomnia scale; ISI, insomnia severity index; THI, tinnitus handicap inventory*.

**Table 3:** The average scores of the patients in the two groups at baseline and 1-week follow up.

	**NBO**			**Control**		
	**Baseline**	**1 week**	***p*-value**	**Baseline**	**1 week**	***p*-value**
VAS	4.70 ± 2.16	2.90 ± 2.03	0.024	3.42 ± 2.39	3.50 ± 2.54	0.754
HIT-6	53.40 ± 12.15	50.30 ± 13.04	0.041	54.17 ± 11.98	55.00 ± 11.36	0.096
HADS	20.60 ± 7.69	19.60 ± 7.44	0.461	20.88 ± 6.33	20.63 ± 6.23	0.351
AIS	12.25 ± 5.05	11.50 ± 5.75	0.121	10.73 ± 3.47	11.36 ± 2.66	0.341
ISI	16.58 ± 8.15	15.33 ± 8.76	0.183	12.00 ± 5.95	12.55 ± 5.72	0.341
THI	50.60 ± 29.72	47.67 ± 32.21	0.172	62.57 ± 32.63	62.00 ± 32.23	0.356

*Headache: VAS and HIT-6; anxiety/depression: HADS; insomnia: AIS and ISI; tinnitus/head noise: THI*.

*NBO, normobaric oxygen; VAS, visual analog scale; HIT-6, Headache Impact Test-6; HADS, hospital anxiety and depression scale; AIS, Athens insomnia scale; ISI, insomnia severity index; THI, tinnitus handicap inventory*.

### 6-Month Follow-Up

26 patients (14 in the NBO group and 12 in the control group) finished the follow-up with good compliance. Eight out of 14 cases (57.1%) in the NBO group reported improvement (three remarkable improvement, five mild improvement, four no change, and two mild deterioration) and one out of 12 patients in the control group reported mild improvement (one mild improvement, seven no change, and four mild deterioration), *p* = 0.014.

### Electroencephalogram Analysis

A total of 10 patients (31.25 ± 8.31 years, 2 males and 8 females) and 21 healthy volunteers (30.57 ± 9.31 years, 4 males and 17 females) completed the EEG recording in this study. Visual evaluation showed paroxysmal slow activities in their EEG maps (Ding J. and Liu Y. evaluated the EEG maps in a blind manner). Further quantitative analysis indicated that the slow-frequency activities increased in the CVOD-related EEG, all of which are detailed in Table 4.

**Table 4:** EEG features in the CVOD patients and the healthy volunteers.

	**CVOD**	**Healthy**	***p*-value**
Case number	10	21	-
**Global area**			
Beta AP	395.74 ± 109.07	477.87 ± 218.70	0.273
Alpha AP	1439.22 ± 810.63	2131.80 ± 1374.09	0.153
Theta AP	534.84 ± 190.43	528.60 ± 222.71	0.940
Delta AP	927.39 ± 290.38	799.74 ± 405.39	0.381
Beta RP	0.13 ± 0.04	0.14 ± 0.10	0.483
Alpha RP	0.40 ± 0.16	0.51 ± 0.12	0.045
Theta RP	0.16 ± 0.04	0.14 ± 0.04	0.091
Delta RP	0.31 ± 0.13	0.21 ± 0.09	0.022
TAR	0.50 ± 0.29	0.29 ± 0.12	0.028
DAR	1.11 ± 1.15	0.46 ± 0.31	0.043
DTABR	1.03 ± 0.63	0.58 ± 0.30	0.035
**Fronto-central area**			
Beta AP	126.63 ± 35.74	125.38 ± 55.92	0.949
Alpha AP	462.06 ± 283.92	550.06 ± 240.96	0.377
Theta AP	201.05 ± 68.32	192.89 ± 76.38	0.776
Delta AP	322.14 ± 95.16	272.75 ± 129.85	0.294
Beta RP	0.12 ± 0.04	0.12 ± 0.07	0.912
Alpha RP	0.38 ± 0.17	0.47 ± 0.11	0.086
Theta RP	0.18 ± 0.04	0.17 ± 0.04	0.381
Delta RP	0.32 ± 0.14	0.24 ± 0.10	0.074
TAR	0.61 ± 0.36	0.39 ± 0.16	0.087
DAR	1.33 ± 1.50	0.58 ± 0.37	0.135
DTABR	1.21 ± 0.82	0.74 ± 0.35	0.186

*EEG, electroencephalogram; CVOD, cerebral venous outflow disturbance; AP, absolute power; RP, relative power; TAR, theta/alpha AP ratio; DAR, delta/alpha AP ratio; DTABR, (delta+theta)/(alpha+beta) AP ratio*.

**Figure 3:**
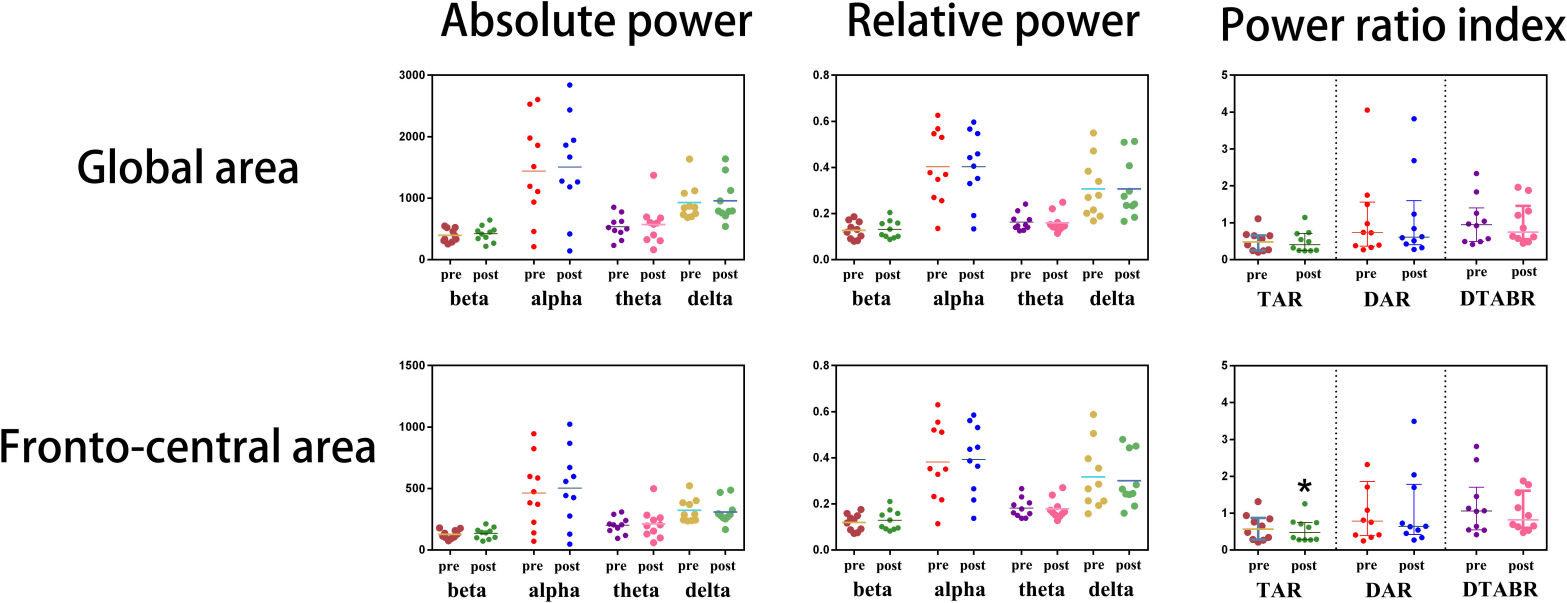
Immediate electroencephalogram (EEG) analysis before and after normobaric oxygen (NBO) intervention.

The impacts of NBO on the abnormal EEG were evaluated in eight out of 10 CVOD patients; two cases were ruled out due to inevitable artifact in the second EEG recordings. After 1 h of NBO intervention, the global slow activities declined, and the fast activities increased compared with the baseline (prior to vs. post-NBO, delta: 0.32 ± 0.14 vs. 0.31 ± 0.14, *p* = 0.756; theta: 0.16 ± 0.04 vs. 0.15 ± 0.03, *p* = 0.098; alpha: 0.39 ± 0.17 vs. 0.40 ± 0.17, *p* = 0.536; and beta: 0.12 ± 0.04 vs. 0.13 ± 0.04, *p* = 0.375), despite no significance. The global TAR, DAR, and DTABR were reduced but with no significance as well (TAR: 0.53 ± 0.31 vs. 0.49 ± 0.32, *p* = 0.242; DAR: 1.25 ± 1.26 vs. 1.25 ± 1.31, *p* = 0.991; and DTABR: 1.11 ± 0.68 vs. 1.02 ± 0.62, *p* = 0.492). As for the fronto-central electrodes, the slow activities declined and the fast activities increased after 1-h NBO performance but did not reach statistical significance as well (delta: 0.33 ± 0.16 vs. 0.31 ± 0.13, *p* = 0.261; theta: 0.18 ± 0.04 vs. 0.17 ± 0.03, *p* = 0.212; alpha: 0.37 ± 0.18 vs. 0.39 ± 0.17, *p* = 0.218; and beta: 0.11 ± 0.04 vs. 0.13 ± 0.05, *p* = 0.168). Notably, the fronto-central TAR was significantly declined after undergoing NBO (0.65 ± 0.38 vs. 0.56 ± 0.35, *p* = 0.030); the DAR and DTABR also tended to be lower post-NBO performance (DAR: 1.50 ± 1.64 vs. 1.20 ± 1.13, *p* = 0.207 and DTABR: 1.31 ± 0.88 vs. 1.06 ± 0.58, *p* = 0.098). All of above are shown in Figure 3.

## Discussion

In this study, we found that NBO might relieve CVOD-related symptoms in a short time, especially for headache, and this effect has potential to be maintained for a long run. This may be due to the correction of CVOD-induced brain dysfunctions presented in the EEG maps afforded by NBO performance. As there are few effective methods to ameliorate CVOD-related symptoms currently, this study provided a novel approach on controlling this refractory disease entity.

After 1-week NBO use, 47.7% of the patients reported that their symptoms attenuated, especially headache relief, whereas at the same period, patients without NBO use complained of symptoms being unchanged or even deteriorated. As previous publications report, CVOD-related non-focal neurological symptoms are classified into three types: the ischemic–hypoxic condition always causes headache, insomnia, dizziness, dry eyes, uncomfortable neck, anxiety, and memory decline; the abnormal collateral vessel nets and the blood outflow turbulence contribute to tinnitus, head noise, and hearing impairment; severe IH leads to massive headache, visual impairment, and fierce vomiting (2–6). NBO is capable of elevating pO_2_ per volume in the brain tissue immediately, which can improve the ischemic–hypoxic condition, so as to ameliorate relevant non-focal neurological symptoms (16). The pO_2_ elevation in the brain tissue is the rationale for the protective effect afforded by NBO on CVOD. Tinnitus or head noise is always considered to be mainly caused by abnormal vessel dilation and blood outflow turbulence, whereby transient NBO may not relieve these symptoms effectively. NBO cannot treat CVOD-related IH as well and is less likely being helpful in visual recovery. In contrast, stenting in stenosis segments is useful in reducing high intracranial pressure (7, 10, 34). After long-term continuous NBO treatment, half of the patients in the NBO group reported that their symptoms ameliorated; despite this, a detailed questionnaire cannot be finished due to the constraints of the phone call follow-up.

In general, the subjective assessment outcomes indicated that NBO might yield some benefit to improve hypoxia-related symptoms within a short period of time but could not get rid of the structural anomaly-related symptoms. The structural anomaly could be modified by endovascular interventions as reported in some previous publications (7, 10, 34, 35). Despite that endovascular stenting is able to restore the venous outflow, some patients with osseous impingement or other external compression induced CVOD cannot gain the benefit from stenting (4). Meanwhile, the surgery is not without risk because a proper stent for the intracranial or extracranial veins is not still invented. NBO may be an appropriate and convenient adjuvant method to relieve CVOD-related symptoms.

Because the aforementioned subjective assessment may be not convincing enough, we select EEG as the powerful objective evidence in this study. EEG is a sensitive approach for detecting cerebral ischemia and hypoxia (36). The abnormal enhancement in the delta and theta activities could be always seen in the ischemic EEG maps. Cerebral venous outflow disturbance-induced CCI may also contain the abnormal increased slow activities (36). Compared with the EEG maps in health, CVOD-related EEG had higher delta and theta RP accompanied with lower alpha and beta RP over the global area of the brain. The RP mainly reflects the components of the EEG oscillations; higher slow-frequency RP means more frequent slow-activity release. Moreover, the TAR, DAR, and DTABR, which represent the ratio of fast activities to slow activities, were significantly higher in the CVOD EEG in comparison with healthy EEG. This further provided more convincing evidences that CVOD could promote the slow-frequency oscillation release and retard the fast-frequency activities. The slow-activity RP and the TAR, DAR, and DTABR were considered to be associated with the neurological outcome in acute stroke patients (37–39). These indices are different from those of the healthy EEG in the CVOD-related EEG that represented brain dysfunctions.

After 1-h NBO intervention, both the global and fronto-central slow activities were suppressed, and the fast activities were restored, however without statistical significance. It is noteworthy that the fronto-central TAR was significantly decreased after undergoing NBO, and the DAR and DTABR were also reduced but without significance. These results indicated that NBO has potential to improve CVOD-related brain dysfunctions, which might further provide objective evidences to verify the efficacy of NBO on CVOD. As the short-term brain functional improvement, we hypothesized that NBO can also be used for patient pre-conditioning, especially prior to endovascular stenting or balloon dilating in the cerebral or jugular venous stenosis. Notably, NBO is capable of promoting exogenous erythropoietin (EPO) production, which plays a pivotal role in anti-apoptosis and neuroprotection in patients with cerebral ischemia (40). Brain functional improvement prior to surgery may enlarge the benefits and decrease the complications in a long run.

There are several limitations that need to be addressed. (1) The sample size was small to reach a statistical significance in some indices. (2) Most of the clinical outcome evaluation methods were based on subjective questionnaires. Despite the blinded manner, the subjective assessors may bias the results toward null hypothesis to some extent. (3) A simple mask was used to inhale oxygen in this study; however, more advanced devices should be used in the future, such as a double-trunk mask, which is easy to use and may produce better results (41). (4) We declared that NBO might only relieve some part of symptoms, especially headache. Surgery is still the ultimate method to reopen the stenosed veins and restore the venous outflow, despite that the evidence and experience are still far from enough.

## Conclusions

NBO may be a safe and promising adjuvant therapy to ameliorate CVOD-related symptoms, especially for headache. It can also correct CVOD-related EEG anomalies in some degree. Considering the aforementioned limitations in this study, a larger sample size and well-designed prospective clinical trial is needed to further verify our conclusions in the future.

## Data Availability Statement

The raw data supporting the conclusions of this article will be made available by the authors, without undue reservation.

## Ethics Statement

The studies involving human participants were reviewed and approved by the Institutional Ethic Committee of Xuanwu Hospital, Capital Medical University (Beijing, China). The patients/participants provided their written informed consent to participate in this study.

## Author Contributions

JD and YL performed all the statistical analyses with the guidance of the Department of Statistics of Capital Medical University and drafted the manuscript. JD generated the random allocation sequence. XL provided suggestions for the study design and revised the manuscript. ZC, JG, KJ, and ZW selected appropriate subjects and conducted the assessments. RM, YD, and XJ reviewed and edited the manuscript. XJ and RM contributed to the conception and design of this study and proposed the amendments. All authors contributed to the article and approved the submitted version.

## Conflict of Interest

The authors declare that the research was conducted in the absence of any commercial or financial relationships that could be construed as a potential conflict of interest.
